# Thymoquinone-Incorporated CollaGee Biomatrix: A Promising Approach for Full-Thickness Wound Healing

**DOI:** 10.3390/pharmaceutics16111440

**Published:** 2024-11-11

**Authors:** Nusaibah Sallehuddin, Looi Qi Hao, Adzim Poh Yuen Wen, Nur Izzah Md Fadilah, Manira Maarof, Mh B. Fauzi

**Affiliations:** 1 Department of Tissue Engineering and Regenerative Medicine, Faculty of Medicine, University Kebangsaan Malaysia, Jalan Yaacob Latif, Cheras, Kuala Lumpur 56000, Malaysia; 2 My Cytohealth Sdn. Bhd., Hive 5, Taman Teknologi, MRANTI, Bukit Jalil, Kuala Lumpur 57000, Malaysia; 3 Department of Surgery, Hospital Canselor Tuanku Muhriz, Jalan Yaacob Latif, Cheras, Kuala Lumpur 56000, Malaysia; 4 Advance Bioactive Materials-Cells UKM Research Group, University Kebangsaan Malaysia, Bangi 43600, Malaysia

**Keywords:** CollaGee, collagen, gelatin, elastin, thymoquinone, wound healing, biomaterial, antibacteria

## Abstract

Wound infection is the leading cause of delayed wound healing. Despite ongoing research, the ideal treatment for full-thickness skin wounds is yet to be achieved. Skin tissue engineering provides an alternative treatment, with the potential for skin regeneration. **Background/Objectives**: Previously, we characterized a collagen–gelatin–elastin (CollaGee) acellular skin substitute and evaluated its cytocompatibility. The assessments revealed good physicochemical properties and cytocompatibility with human dermal fibroblasts (HDF). This study aimed to incorporate thymoquinone (TQ) as the antibacterial agent into CollaGee biomatrices and evaluate their cytocompatibility in vitro. **Methods**: Briefly, dose–response and antibacterial studies were conducted to confirm the antimicrobial activity and identify the suitable concentration for incorporation; 0.05 and 0.1 mg/mL concentrations were selected. Then, the cytocompatibility was evaluated quantitatively and qualitatively. **Results**: Cytocompatibility analysis revealed no toxicity towards HDFs, with 81.5 + 0.7% cell attachment and 99.27 + 1.6% cell viability. Specifically, the 0.05 mg/mL TQ concentration presented better viability, but the differences were not significant. Immunocytochemistry staining revealed the presence of collagen I, vinculin, and alpha smooth muscle actin within the three-dimensional biomatrices. **Conclusions**: These results suggest that TQ-incorporated CollaGee biomatrices are a promising candidate for enhancing the main key player, HDF, to efficiently regenerate the dermal layer in full-thickness skin wound healing. Further investigations are needed for future efficiency studies in animal models.

## 1. Introduction

Skin wounds are classified into three categories according to their depth, which are superficial, partial-thickness, and full-thickness skin wounds [1]. Superficial skin wounds solely affect the epidermis such as abrasions or scratches. Partial-thickness skin wounds affect both the epidermis and dermis such as lacerations and ulcers. Lastly, full-thickness skin wounds affect all layers of skin and may also reach the muscles and bones such as gunshots or incision wounds [2]. Wounds that are larger than 10% of a person’s skin are life-threatening [3]. Due to various causes such as shortage of cell sources, excessive extracellular fluid loss, constrained blood supply, immunodeficiency, metabolic illness, various environmental conditions, and added complications such as infections, full-thickness skin wounds have a poor ability to heal and regenerate [4]. Most full-thickness skin wounds heal by leaving scars [5]. A full-thickness skin wound therefore requires immediate management to achieve complete healing with minimal scarring and maintain the skin’s functionality and integrity.

Split-thickness skin grafts (STSGs) continue to be the gold standard treatment for full-thickness wound management, despite substantial research in this area [6]. Autologous grafts do, however, have several drawbacks, including immunogenicity and a limited supply. STSGs may also cause hypertrophic scarring, diminished functionality, and an unsightly appearance [7]. Technology for wound management therefore focuses on facilitating and improving the physiological process. Skin tissue engineering aims to rebuild skin tissue at the site of a wound using a biomatrix, body cells, and growth factors [8]. Typically, a biomatrix is a porous three-dimensional (3D) structure that provides an appropriate microenvironment for regenerating tissues and organs [9]. Cellular skin substitutes (CSSs) are a cytocompatible biomatrix and can improve angiogenesis; however, product storage is still a problem. Acellular skin substitutes (ASSs), fortunately, could be utilized to get over this problem because they are substantially less expensive, can be kept on the shelf for a longer time, and can be used at any time by doctors or surgeons. ASS objectives are to maintain moisture, absorb exudate, and guard against infection. They have been utilized to treat both acute and chronic wounds, despite the fact none of the current available ASSs have the optimal parameters for wound healing [10].

ASSs were originally used to treat superficial wounds and burns in 1970 [11]. Since then, the technology for ASSs has developed quickly. ASS products like Alloderm, Dermalon, Permacol, and Integra range from the decellularized dermis to 3D biomatrices and can be derived from natural or artificial sources. In order to treat deep partial- and full-thickness burn wounds, Alloderm, which is made from de-epithelialized cadaveric skin, has been frequently utilized [12]. However, Permacol, an acellular cutaneous xenograft, is not recommended because the biomatrix is chemically crosslinked and the cytocompatibility outcomes are subpar [13]. Integra, a skin tissue engineering template, is made of a lower porous layer of collagen and glycosaminoglycan and an upper layer of silicon [14]. Nevertheless, although there is a lot of research being conducted to design ASSs, there is not yet a single product that satisfies all the standards for optimum wound healing [15].

Wound infection may result in a low-grade inflammatory response and delayed wound healing. Hence, bacterial invasion must be prevented. The human microbiome is an ecosystem of bacteria that naturally inhabits human skin and contains roughly 10^12^ microorganisms [16]. Human microbiome composition can be affected by age, antibiotic use, environment, nutrition, and other factors [17]. Infection can develop when the skin is injured or when there is an imbalance between the human microbiome and pathogenic bacteria [18]. A contaminated wound means the presence of a non-proliferating human microbiome in the wound area. Colonization of the wound happens if the environment around the wound is favorable for bacterial growth [19]. In the beginning, the wound is colonized by Gram-positive bacteria. This colonization does not cause a significant immune reaction. Therefore, there are no clinical signs of infection. However, in the later stage, the wound will eventually be colonized by Gram-negative bacteria. This colonization triggers a limited immunological reaction with clinical signs of infection [20]. Consequently, a possible approach of authentic ASSs must possess antibacterial properties to ensure a low risk of severe infection and optimal wound healing [21].

Previously, we fabricated collagen-gelatin-elastin (CollaGee) biomatrices as a potential ASS using the lyophilization method and investigated the physicochemical, mechanical, and cytocompatibility properties. CollaGee crosslinked with genipin (GNP) was revealed to have good characterization (porosity of more than 50%, pore size range of 100 to 200 µm, swelling ratio of more than 1000%, resilience and crosslinking degree of more than 50%, modulus of more than 1.0 GPa) and non-cytotoxicity towards human dermal fibroblasts (HDF)s with sustained viability of more than 70% [22]. As CollaGee does not have antibacterial properties, CollaGee can be combined with antibacterial agents such as herbal components to offer an antibacterial function. Herbal components are preferred over antibiotics to avoid antibiotic abuse and resistance issues [23]. Ramanathan et al. (2017) reported that collagen/*Coccinia grandis* biomatrices have good biocompatibility and antibacterial activity against *Escherichia coli* and *Staphylococcus aureus* and improved wound healing in vivo [24].

Since ancient times, *Nigella sativa* has been used as a traditional medicine to treat skin wounds [2]. Thymoquinone (TQ) is the main active ingredient in NS and is responsible for the wound healing properties of NS [25]. TQ has anti-inflammatory, antioxidant, antibacterial, and angiogenic properties that facilitate wound healing along with promoting proliferation and granulation tissue formation [26]. Selçuk et al. (2013) compared the effects of silver sulfadiazine (SS) and TQ on wound healing and discovered that TQ promoted faster wound healing than the current gold standard therapy, SS [27]. According to Negi et al. (2020), TQ promotes re-epithelialization, facilitates anti-inflammatory response, reduces oxidative stress, and increases HDF formation and granulation tissue development [28]. Furthermore, TQ treatments also successfully improved total antioxidant status and reduced total oxidant status levels [2].

Although there have been significant advancements in wound healing research and the development of ASS, there is still a need to address several gaps in existing knowledge. Despite ongoing research, there is no single ASS product that currently meets all the ideal criteria for optimum healing. There is a need to develop an ASS that is resistant to infection, lacking in antigenicity, cost-effective, readily available, durable, stable, and moisture-retaining, with a wide range of wound coverage. Collagen, which is the most abundant protein in ECM, can be extracted from bovine and porcine tissues. However, this comes with concerns about infection and religious constraints. Exploring alternative sources such as ovine can help overcome these limitations and enhance the effectiveness of ASSs. Previously, we successfully fabricated CollaGee as a potential ASS with good physicochemical, mechanical, thermal, and cytocompatibility properties. The major concern in wound healing is infection, which is the most common cause of wound healing failure. Integrating antibacterial agents such as TQ into ASSs can help prevent infection and improve wound bed conditions. TQ has antibacterial, anti-inflammatory, and antioxidant properties, which could accelerate wound healing. However, the toxicity of TQ in skin cells needs further evaluation. In this study, TQ was incorporated into CollaGee biomatrices, and the cytocompatibility was evaluated both quantitatively and qualitatively. It is essential to understand the cytocompatibility of skin cells with implanted TQ-incorporated biomatrices at appropriate concentrations to achieve dual bioeffects, ensuring successful cell attachment, proliferation, and tissue regeneration.

## 2. Materials and Methods

This study protocol was approved by the Universiti Kebangsaan Malaysia (UKM) Research Ethics Committee (code no. JEP-2019-424).

### 2.1. Dose–Response of TQ

As TQ is hydrophobic, TQ needed to be dissolved first before treatment on HDFs. TQ can be dissolved in dimethyl sulfoxide (DMSO) at a concentration of 14 mg/mL [29,30]; hence, 14 mg/mL TQ (Sigma-Aldrich, St. Louis, MO, USA) in DMSO (Sigma-Aldrich, St. Louis, MO, USA) was prepared. Then, seven concentrations of TQ in F12:DMEM (Gibco, Waltham, MA, USA) containing 10% fetal bovine serum (FBS) (Sigma-Aldrich, St. Louis, MO, USA) were prepared using a serial dilution method from 1 mg/mL. The concentrations were 1, 0.5, 0.25, 0.125, 0.0625, 0.0312, and 0.0156 mg/mL. Briefly, HDFs were seeded in 48-well plates for 24 h. Then, HDFs were treated with different concentrations of TQ for another 24 h. After that, a 3-[4,5-dimethylthiazol-2-yl]-2,5 diphenyl tetrazolium bromide (MTT) (Invitrogen, Waltham, MA, USA) assay was performed to evaluate the cell viability after treatment with TQ. Experiments were performed with six different samples in triplicate. The formula for cell viability (%) was as follows:

(1) $$Cell\;viability\;\left(\%\right)=\frac{A^{t}\;}{A^{f}\;}\times 100$$

where A^t^ is the absorbance of cells treated with TQ and A^f^ is the absorbance of cells in F12:DMEM supplemented with 10% FBS.

### 2.2. Antibacterial Study of TQ

The prepared TQ and positive control gentamicin (Sigma Aldrich, St. Louis, MO, USA) solutions of different concentrations (0.5, 0.25, 0.125, 0.0625, 0.0312, and 0.0156) mg/mL were tested for antibacterial activity against the Gram-positive bacteria *Staphylococcus aureus* (ATCC 25923) and *Bacillus cereus* (SBMW1) and the Gram-negative bacteria *Escherichia coli* (ATCC 25927) and *Pseudomonas aeruginosa* (PA14) using the well diffusion method to measure the zones of inhibition and using a microbroth dilution assay to determine the minimum inhibitory concentrations (MICs).

Regarding the measurement of the zones of inhibition, the bacterial cultures were inoculated in Mueller Hinton Broth (MHB) (Sigma Aldrich, St. Louis, MO, USA) and incubated at 37 °C for 3 h under aerobic conditions. The turbidity of the inoculum was consequently adjusted using MHB to be equivalent to 0.5 McFarland’s standard. Then, 100 µL of TQ and gentamicin solutions were introduced into 5 mm wells prepared with Mueller Hinton Agar (MHA) (Sigma- Aldrich, St. Louis, MO, USA) on plates (inoculated with each bacterium separately) and then the plates were incubated at 37 °C for 24 h; after 24 h, the zone of inhibition was measured.

Meanwhile, regarding the determination of MICs, 100 µL of bacterial inoculum, TQ, and gentamicin solution of different concentrations and sterile water were pipetted to each well of a 96-well plate. The plate was incubated at 37 °C for 24 h. The MIC value was defined as the lowest concentration of the tested agent that could inhibit bacterial growth, observed as a lack of turbidity in growth media. Both studies were performed in six sets of samples in triplicate. The formula for the absorbance of the sample that inhibited bacteria was as follows:

(2) $$Absorbances\;of\;sample\;inhibits\;bacteria\;\left(AU\right)=A^{sba}-A^{sbr}$$

while the formula for the absorbances of the positive control that inhibited bacteria as follows:

(3) $$Absorbances\;of\;positive\;control\;inhibits\;bacteria\;\left(AU\right)=A^{pba}-A^{br}$$

where A^sba^ is the absorbance of samples with the presence of bacteria, A^sbr^ is the absorbance of samples in broth only, A^pba^ is the absorbance of the positive control with the presence of bacteria, and A^br^ is the absorbance of broth only.

### 2.3. Collagen Type I Extraction and Purification

Collagen was isolated from ovine tendon by the acetic acid dissolution method. The ovine tendon was immersed in 0.35 M acetic acid (MERCK, Darmstadt, Germany) for 72 h to dissolve the collagen protein and remove any impurities. The swollen tendons were blended with sterilized acetic acid to produce a homogenous solution at a chilling temperature of 4–8 °C [31]. Then, 40 mL of the solution was put into a 50 mL tube using a serological pipette. Next, 2 g of sodium chloride (Sigma-Aldrich, St. Louis, MO, USA) was added to each tube and chilled for 24 h. Then, the mixture was centrifuged at 5000 rpm (Hettich Zentrifugen, Fohrenstrabe, Tuttlinhen, Germany) for 10 min at 4 °C. The supernatant was collected and further purified by dialyzing in a dialysis tube for 72 h. After dialysis, the collagen precipitates were collected and poured into a Petri dish and frozen in a −20 °C freezer before freeze drying for 24 to 48 h. Lastly, the extracted collagen was redissolved in 0.35 M sterilized acetic acid to make a collagen stock.

### 2.4. Fabrication of TQ-Incorporated CollaGee Sponges

Two concentrations of TQ were selected from the dose–response and antibacterial experiments. The sponge was fabricated by mixing 1.425% (*w*/*v*) collagen stock and 0.7% (*w*/*v*) gelatin solution at a volume ratio of 9:1. The mixtures were stirred using a magnetic stirrer for 5 min. Then, 10 mg of elastin powder was added and further mixed for 10 min until the powder dissolved. After that, initial concentrations of 250 µL of TQ in DMSO were gently stirred with the mixtures to obtain the intended concentrations (0.05 and 0.1 mg/mL). Next, the mixtures were poured into the desired mold. Then, the plate was frozen in a −80 °C freezer for 6 h and freeze-dried for 24–48 h at −56 °C and 5 mTorr of atmospheric pressure. After that, the crosslinking was performed by immersion in a 0.1% (*w*/*v*) GNP (Fujifilm Wako Pure Chemical Corporation, Shibukawa, Japan) solution for 6 h at room temperature. The GNP solution was made by mixing the crystallized powder in 70% ethanol (EtOH; MERCK, Darmstadt, Germany) at room temperature. Then, the sponges were rinsed with Dulbecco’s Phosphate Buffered Saline (DPBS) (Sigma-Aldrich, St. Louis, MO, USA) three times for 10 min each to obtain CollaGee_GNP, CollaGee0.05%_GNP, and CollaGee0.1%_GNP. Non-crosslinked groups were used as control, namely, CollaGee, CollaGee0.05%, and CollaGee0.1%. The crosslinked sponges were then freeze-dried for 24–48 h for further analysis.

#### 2.4.1. Gross Appearance

The gross appearance of the fabricated biomatrices was captured with a digital camera (Nikon, Tokyo, Japan).

#### 2.4.2. Chemical Characterization

The chemical characterization was performed using Fourier transform infrared spectroscopy (FTIR). Spectral data of the biomatrices were obtained using a PE spectrum 100 FTIR spectrometer (PE, Waltham, MA, USA) across a wavelength range of 700–4000 cm^−1^. The absorbance peaks were examined to identify the chemical structure and assess any chemical changes resulting from the crosslinking process. Additionally, energy dispersive X-ray (EDX) (Merlin, ZEISS, Jena, Germany) analysis was performed to determine the elemental composition within the fabricated biomatrices and provide quantitative data.

### 2.5. Isolation and Culture of HDFs

HDFs were isolated from redundant tissue obtained from six consenting patients and processed as previously described by Fauzi et al. (2019) [31]. In brief, 3 cm^2^ of skin samples were cleaned, minced, and digested using a two-step enzymatic process involving 0.6% collagenase type I (Worthington, Columbus, OH, USA) at 37 °C for 4–6 h followed by treatment with trypsin-EDTA (Sigma-Aldrich, St. Louis, MO, USA) for 10 min. The resulting cell suspension was centrifuged and resuspended in a co-culture medium consisting of an equal amount of Epilife (Gibco, Waltham, MA, USA) and F12:DMEM supplemented with 10% FBS. The suspensions containing human epidermal keratinocytes (HEK) and HDFs were seeded into a polystyrene culture plate and incubated at 37 °C with 5% carbon dioxide. The culture medium was replaced three times per week. Once the cells reached 70–80% confluency, HDFs were isolated by differential trypsinization and cultured in a 75 cm^2^ T-flask with F12:DMEM supplemented with 10% FBS.

#### 2.5.1. Cell Toxicity Assessment

The LIVE/DEAD^®^ Cell Viability Assay (Invitrogen, Waltham, MA, USA) was used to analyze the cytotoxic effect of the biomatrices. The procedure was performed according to the manufacturer’s protocol. The biomatrices were sterilized using 70% EtOH for 30 min and then washed with phosphate-buffered saline (PBS) (Sigma–Aldrich, St. Louis, MO, USA) before being dried overnight. After that, 20 µL of cell suspension containing 1 × 10^5^ HDFs were cultured directly on the surface of the sterilized biomatrices at five different spots and incubated in a 37 °C incubator with 5% CO_2_ for 24 h. After that, the culture medium was removed and the biomatrices were washed with PBS. Then, the biomatrices were incubated in the same condition with 2 mM calcine acetomethoxy calcein derivate (AM) and 4 mM ethidium homidimer-1 (EthD-1) in F12:DMEM for 30 min and washed with DPBS. Afterwards, F12:DMEM was added to the sponges again. Subsequently, observation of the cultures was performed using Nikon A1R-A1 confocal laser scanning microscopy (CLSM) (Nikon A1R-A1, Nikon, Tokyo, Japan). The live cells were stained with a green color while the dead cells were stained with a red color.

The morphological features of the cells on the biomatrices were observed using a scanning electron microscope (SEM) (Nikon, Tokyo, Japan). Cell-seeded biomatrices were washed with PBS before fixing with 4% paraformaldehyde at 4 °C for 24 h. After that, the biomatrices were washed three times with milli-Q water. Subsequently, the biomatrices underwent serial dehydration with 35%, 50%, 75%, and 95% EtOH for 10 min each and 100% EtOH 3 times for 10 min each. Lastly, the biomatrices were dried overnight using a critical point dryer (CPD 030; Bal-Tec, Los Angeles, CA, USA) and subsequently coated with nanogold before viewing under a SEM [31].

#### 2.5.2. Cell Viability and Attachment Evaluation

The cytocompatibility of the 3D biomatrices was determined by MTT assay via the direct method. Briefly, 5 mm^2^ biomatrices were placed in 96-well plates and sterilized using 70% EtOH for 30 min and then washed with PBS before being dried overnight. After that, 20 µL of cell suspension containing 3 × 10^4^ HDFs were directly seeded on the surface of the biomatrices. The plates were incubated for 2 h before adding 100 µL of culture medium to the wells. Finally, the cell culture plates were incubated for 1, 3, and 7 days separately. After the designated periods, the cell culture medium was removed and the biomatrices were gradually washed with PBS. Afterwards, 300 µL F12: DMEM and 30 µL MTT solution (5 mg/mL) in PBS were added to each sample followed by incubation for 4 h at 37 °C for MTT formazan formation. Then, 225 µL of the medium was discarded from each well and washed with PBS. After that, a volume of 100 µL DMSO was added to each well to dissolve the formazan crystals and incubated at 37 °C for 20 min. Then, the solutions were transferred from each well to other 96-well plates. The absorbances were measured at a 540 nm wavelength using a microplate reader (Power Wave XS; Bio-TEK Instruments, Winooski, VT, USA). Negative controls were made by adding 300 µL of F12:DMEM to 30 µL of MTT solution without cells. The positive control for this analysis was the viability of cells on the 2D cell culture plate. Hence, the viability of cells on the 3D biomatrices was compared to the viability of cells on the 2D cell culture plate. The formula for cell viability (%) was as follows:

(4) $$Cell\;viability\;\left(\%\right)=\frac{A^{t}}{A^{u}}\times 100\;$$

where A^t^ is the absorbance of the solution of the cells seeded on the biomatrices and A^u^ is the absorbance of the solution of the cells seeded on the well plate.

The cell attachment assay of HDFs on the 3D biomatrices was determined by a Trypan blue dye exclusion assay. In short, 5 × 10^5^ HDFs were cultured on the biomatrices and incubated for 24 h. Then, the culture media were collected and centrifuged for 5 min at 5000 rpm, after which, the supernatant was discarded and the pellet was resuspended in 5 mL of DPBS. A viable cell count of the unattached cells was performed by mixing them with Trypan blue (Sigma-Aldrich, St. Louis, MO, USA) and counting them in a hemocytometer (Optik Labor, 0.100 mm, Gorlitz, Germany) under a light microscope (Olympus CK40, Tokyo, Japan). Both experiments were performed in six sets of samples in triplicate. The number of cells in DPBS referred to unattached cells. Hence, to measure the attached cells on the biomatrices, the number of unattached cells needed to be subtracted from the number of initial cells seeding. The formula for cell attachment (%) was as follows:

(5) $$Cell\;attachment\;\left(\%\right)=\frac{N^{i}-N^{d}}{N^{i}}\times 100$$

where N^i^ is the number of initial cells seeding and N^d^ is the number of cells in DPBS.

#### 2.5.3. Protein Expression Study

To verify and prove the presence of HDFs within the 3D biomatrices, the sponges were incubated with a primary antibody against collagen-I (Col-I), vinculin, and alpha smooth muscle actin (α-SMA) in a 1:1000 dilution. The primary antibodies were Rabbit Anti-Collagen I (Sigma-Aldrich, St. Louis, MO, USA, Cat# SAB4500362, RRID:AB_10743808), Rabbit Anti-Vinculin (Sigma-Aldrich, St. Louis, MO, USA, Cat# SAB4503069, RRID:AB_10746313), and Rabbit Anti-Alpha Smooth Muscle Actin (Sigma- Aldrich, St. Louis, MO, USA, Cat# SAB5700835), respectively. The biomatrices were seeded with HDFs for 24 h and immunostaining was performed after fixation using 4% paraformaldehyde (Sigma-Aldrich, St. Louis, MO, USA). Before staining, the cell-seeded biomatrices were rinsed three times with PBS and then permeabilized using a 0.5% Triton X-100 (Sigma-Aldrich, St. Louis, MO, USA) solution for 20 min. After the biomatrices were rinsed again three times in PBS, the biomatrices were incubated in 10% goat serum (Sigma-Aldrich, St. Louis, MO, USA) in PBS for 1 h. A secondary antibody, Anti-Rabbit IgG (H + L), F (ab’)2 fragment, CF^TM^488A antibody (Sigma-Aldrich, St. Louis, MO, USA, Cat# SAB4600389, RRID:AB_2917980), was used to identify the primary antibody. Cell nuclei were stained with 4′,6-diaminido-2-phenylindole (DAPI) (Invitrogen, Waltham, MA, USA) in a 1:15,000 dilution. Cell-seeded biomatrices were then rinsed and viewed under CLSM. Fluorescent photomicrographs were taken of representative areas.

### 2.6. Statistical Analysis

The statistical analysis was conducted using GraphPad Prism (Version 8.0; GraphPad Software Inc., San Diego, CA, USA). Numerical data were presented as the mean + standard deviation. Statistical significance was evaluated by one-way or two-way analysis of variance (ANOVA) followed by post-hoc Tukey’s multiple comparison tests, with *p* < 0.05 values considered significant.

## 3. Results

### 3.1. Dose–Response of TQ

**Figure 1:**
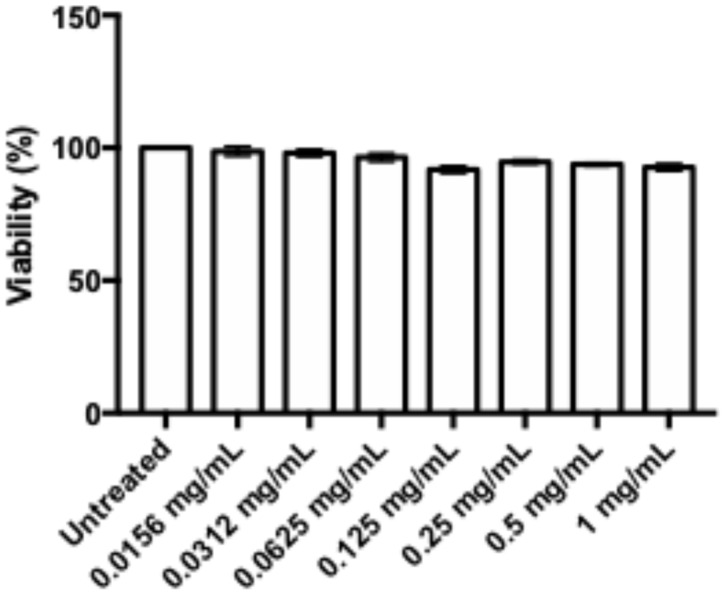
Dose–response of TQ in HDF.

HDF viability with TQ treatments is shown in Figure 1. The viability of HDFs with TQ concentrations of 0.0156, 0.0312, 0.0625, 0.125, 0.25, 0.5, and 1 mg/mL were 98.52 ± 0.92%, 98.12 ± 0.93%, 96.39 ± 1.43%, 91.78 ± 0.93%, 94.74 ± 0.67%, 93.76 ± 0.49%, and 92.70 ± 0.92%, respectively. There were no significant differences in the viability of HDFs among all groups.

### 3.2. Antibacterial Analysis of TQ and Determination of MIC

**Figure 2:**
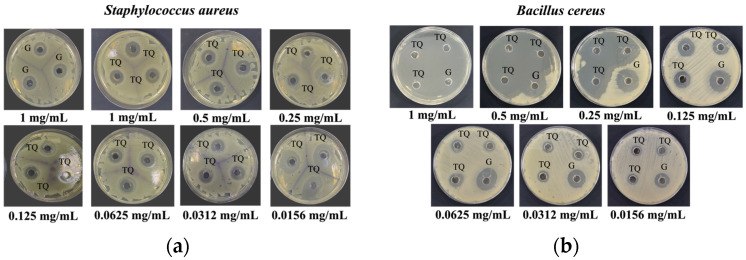

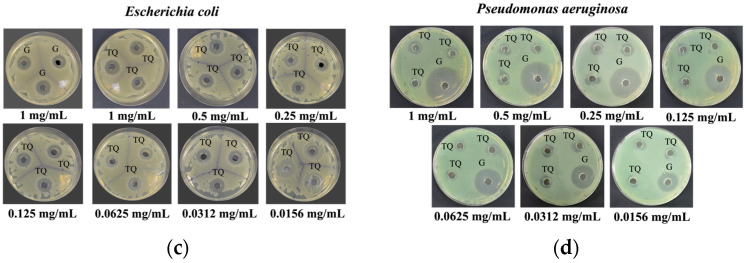
Gross appearances of zones of inhibition of TQ against (**a**) *Staphylococcus aureus*, (**b**) *Bacillus cereus*, (**c**) *Escherichia coli*, and (**d**) *Pseudomonas aeruginosa*. TQ: Thymoquinone, G: Gentamicin.

**Figure 3:**
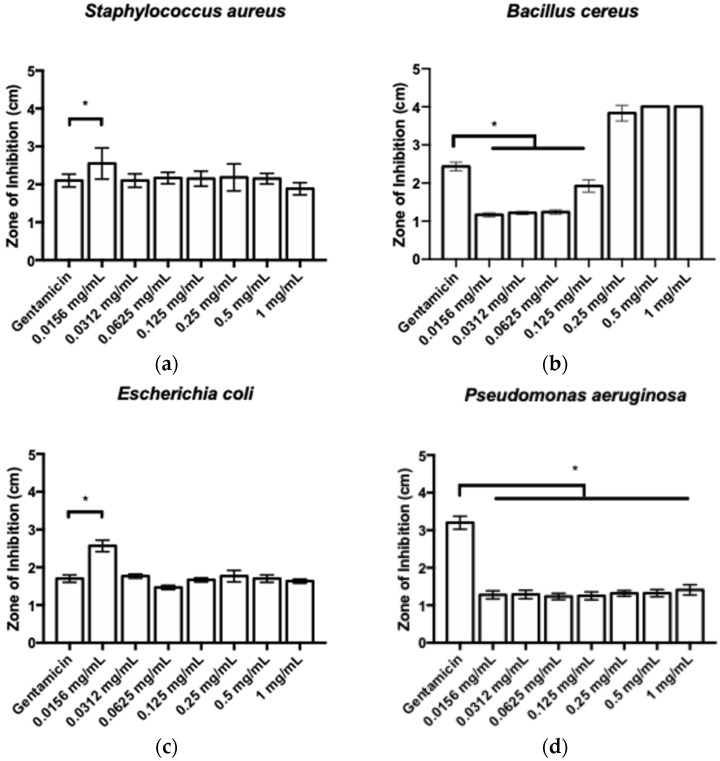
Zones of inhibition of TQ against (**a**) *Staphylococcus aureus*, (**b**) *Bacillus cereus*, (**c**) *Escherichia coli*, and (**d**) *Pseudomonas aeruginosa*. * *p* < 0.05 compared to the control (gentamicin).

The gross appearances of the zones of inhibition of TQ against the Gram-positive bacteria *Staphylococcus aureus* and *Bacillus cereus* and the Gram-negative bacteria *Escherichia coli* and *Pseudomonas aeruginosa* are illustrated in Figure 2. The respective measurements can be observed in Figure 3. Concerning Gram-positive bacteria, TQ concentrations of 0.25, 0.5, and 1 mg/mL exhibited the most substantial zones of inhibition against *Bacillus cereus*, surpassing gentamicin by a significant margin. In contrast, for *Staphylococcus aureus*, a TQ concentration of 0.0156 mg/mL displayed the greatest zone of inhibition, also significantly larger than that of gentamicin. In relation to Gram-negative bacteria, a TQ concentration of 0.0156 mg/mL showed the greatest zone of inhibition against *Escherichia coli,* significantly larger than that of gentamicin. For *Pseudomonas aeruginosa*, a TQ concentration of 1 mg/mL demonstrated the greatest zone of inhibition. Nevertheless, the zone of inhibition of gentamicin was significantly greater than for all TQ concentrations. The MIC results are presented in Table 1. The broth microdilution assay revealed that the MIC values of TQ were 0.0625 mg/mL for both Gram-positive bacteria and 0.125 mg/mL for both Gram-negative bacteria.

### 3.3. Gross Appearances and Chemical Characterization

**Figure 4:**
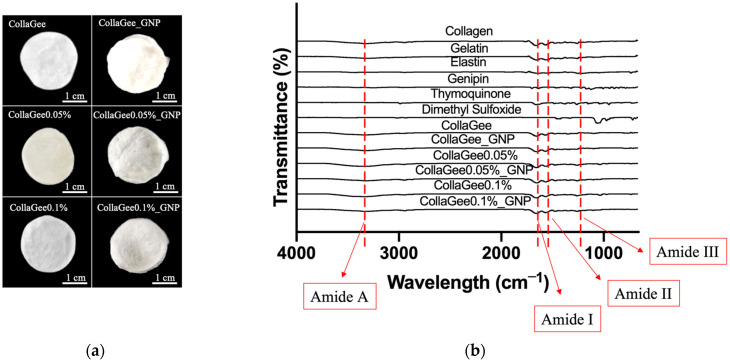
(**a**) Gross appearances of TQ-incorporated CollaGee biomatrices. (**b**) FTIR analysis of CollaGee biomatrices.

The gross appearances of non-crosslinked and crosslinked biomatrices appeared white in color, as shown in Figure 4a. The FTIR spectra of all biomatrices showed similar absorbance peaks, corresponding to Amide A (3500–3100 cm^−1^), Amide 1 (1634–1618 cm^−1^), Amide II (1551–1531 cm^−1^), and Amide III (1285–1231 cm^−1^). No significant shifts were observed in the FTIR spectra following GNP modification, as illustrated in Figure 4b. EDX analysis of the electron images of the biomatrices revealed the presence of three primary elements—carbon, oxygen, and nitrogen—which are the major components of proteins, as shown in Table 2. Sulfur was found in trace amounts.

### 3.4. Cell Attachment, Viability, and Cytocompatibility

**Figure 5:**
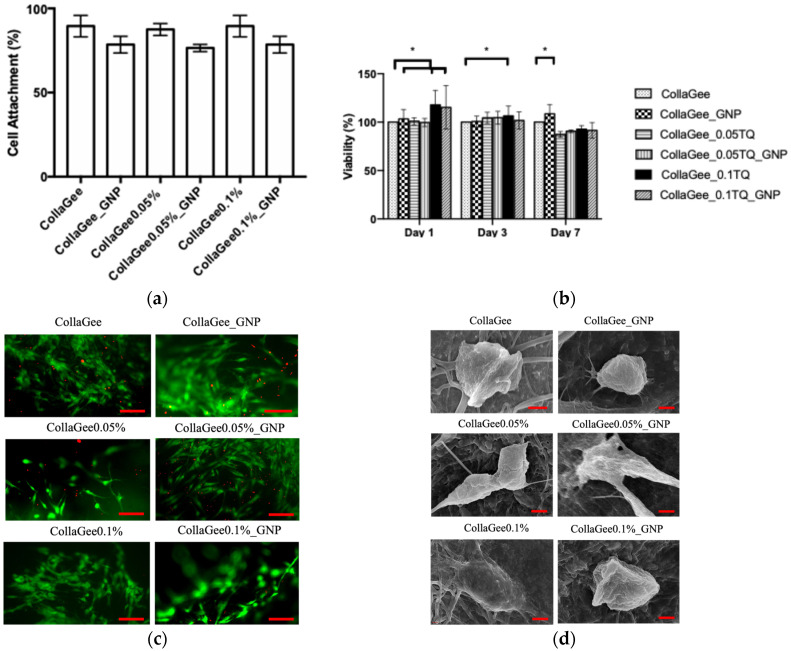
(**a**) Cell attachment assay. (**b**) MTT assay, * *p* < 0.05 compared to the control (CollaGee). (**c**) LIVE/DEAD^®^ assay showing live cells (green) and dead cells (red), scale bar: 100 μm. (**d**) SEM micrographs (scale bar: 1 μm) of HDFs for the biomatrices.

The results of the cell attachment assay of HDFs for the biomatrices via the direct method are given in Figure 5a. The crosslinked groups reported a percentage of cell attachment of 81.50 + 0.71% for CollaGee_GNP, 76.33 + 0.58% for CollaGee0.05%_GNP, and 78.11 + 2.69% for CollaGee0.1%_GNP. The non-crosslinked groups reported a percentage of cell attachment of 93.33 + 0.58% for CollaGee, 87.22 + 1.64% for CollaGee0.05%, and 89.00 + 3.74% for CollaGee0.1%. The biomatrices provided a good attachment surface for HDFs, with cell attachment of more than 70%. The graphs of cell viability for the biomatrices are shown in Figure 5b. The non-crosslinked and non-incorporated group was selected as the control. CollaGee had significantly lower viability compared to CollaGee_GNP, CollaGee0.05%_GNP, and CollaGee0.1%_GNP on day 1; CollaGee0.05% on day 3; and CollaGee_GNP on day 7. Nevertheless, all groups showed viability of more than 80% on all experimental days.

In vitro cytocompatibility of the biomatrices was investigated by LIVE/DEAD^®^ assay using HDFs. The biomatrices were examined with fluorescence images after culture for 24 h and revealed homogeneous cell distribution and high cell viability, as shown in Figure 5c. Live cells were stained in green and dead cells were stained in red. The cells were characterized by an elongated spindle shape. SEM analysis revealed that HDFs seeded onto the biomatrices exhibited a spindle-shaped morphology with excellent attachment. Extensions of the cytoskeleton were observed, along with the presence of released matrices, as shown in Figure 5d.

### 3.5. Protein Expression Analysis

**Figure 6:**
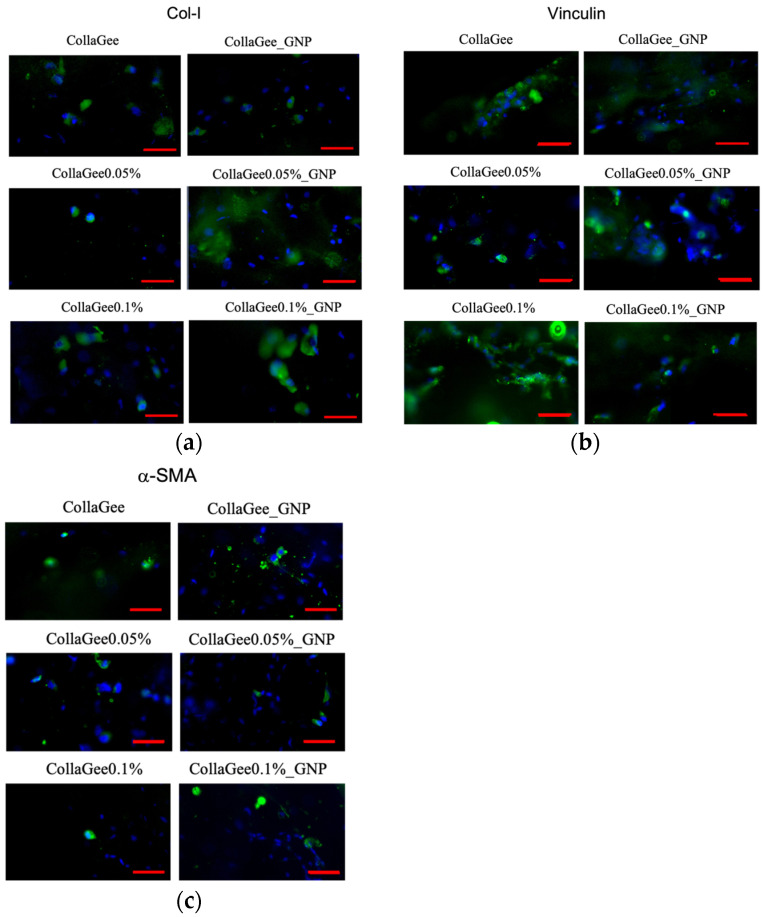
Micrographs of HDFs labeled with (**a**) anti-collagen I (Col-I) antibody (green), (**b**) anti-vinculin antibody (green), and (**c**) anti alpha smooth muscle actin (α-SMA) antibody (green). The blue color (DAPI) represents the nucleus of the HDF. Scale bar: 50 μm.

Immunocytochemistry was performed to identify Col-I, vinculin, and α-SMA in the biomatrices. Fluorescence staining with DAPI and the secondary antibody clearly revealed the expressions of Col-I (Figure 6a), vinculin (Figure 6b), and α-SMA (Figure 6c) within the 3D biomatrices. In addition, HDFs demonstrated round to spindle-shaped morphology, with relatively ununiform distribution across the biomatrices.

## 4. Discussion

The objective of skin tissue engineering is to regenerate skin tissue in the area of a wound by utilizing a combination of biomatrices, cells, and growth factors [8]. While CSSs are cytocompatible and promote angiogenesis, issues related to product storage remain. The utilization of ASSs can address this concern by providing a relatively cheaper alternative that can be stored on shelves for extended periods and used conveniently by clinicians or surgeons. ASSs employ natural or synthetic biomaterials to offer temporary or permanent coverage for skin wounds. The purpose of ASSs is to absorb exudates, maintain moisture, and safeguard wounds against infection. The initial report on ASSs for treating superficial wounds and burns was created in the 1970s [11], after which, ASS technologies have experienced rapid advancements. Nathoo et al. (2014) identified essential characteristics for an ideal skin substitute, including infection resistance, absence of antigenicity, cost-effectiveness, easy availability, durability, stability, moisture retention capability, and broad wound coverage [32]. Despite ongoing research efforts to develop ASSs, there is currently no single product that fulfills all the ideal criteria for optimal wound healing [15].

In our previous study, we developed a CollaGee biomatrix composed of collagen, gelatin, and elastin, which was subsequently crosslinked with GNP. We had previously conducted and published the physicochemical, mechanical, and thermal characterization of these CollaGee biomatrices. The results demonstrated that the biomatrices exhibited good physicochemical characteristics, including more than 50% porosity, pore sizes between 100 and 200 μm, and a swelling ratio greater than 1000%. Moreover, the biomatrices displayed favorable mechanical properties, with resilience and crosslinking degrees exceeding 60%, as well as a modulus higher than 1.0 GPa. Furthermore, cytocompatibility testing conducted on the biomatrices demonstrated minimal toxicity and sustained cell viability towards HDFs [22]. We postulated that the physicochemical and thermal characterizations would have no significant differences following the incorporation of TQ at a small concentration of 0.05 to 0.1% in the biomatrices. A study conducted by Singh et al. (2022) reported no significant changes in porosity observed in decellularized goat small intestine submucosa biomatrices, despite increasing concentrations of curcumin of more than 0.1% [33]. Similarly, Suksaeree et al. (2015) found that the swelling ratio and porosity of chitosan/hydroxypropyl methylcellulose biomatrices did not exhibit any notable differences after the addition of cassumunar oil [34]. Likewise, Jiménez et al. (2022) reported no significant difference in the degradation kinetics of col-I biomatrices when incorporating 0.1% of aloe vera extract [35]. Based on the previous findings, we hypothesized that the physical properties would not significantly vary following the incorporation of 0.1% and 0.05% of TQ into their biomatrices. Nonetheless, we conducted chemical characterization to investigate any changes in peaks or the presence of additional elements resulting from the incorporation of TQ.

**Figure 7:**
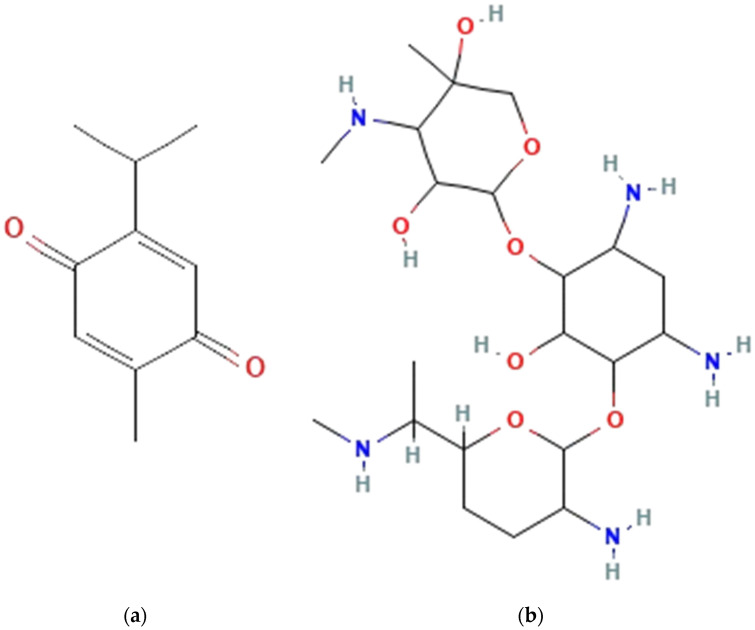
The schematic structure of (**a**) TQ and (**b**) gentamicin. The figures were sourced from the National Centre of Biotechnology Information, PubChem Compound Summary.

TQ (molecular formula: C_10_H_12_O_2_) belongs to the 1,4-benzoquinones class, where hydrogens on the 1,4-benzoquinone ring are substituted with methyl and isopropyl groups, respectively [36]. Gentamicin (molecular formula C_21_H_43_N_5_O_7_) is a common antibacterial that is effective against both Gram-positive and Gram-negative bacteria [37]. Gentamicin is widely used in various types of infections and, hence, was selected as the control in the antibacterial studies. The chemical formulas for TQ and gentamicin are shown in Figure 7. Seven concentrations of TQ were prepared using the serial dilution method from 1 mg/mL. Based on the dose–response analysis, TQ was found to induce cell proliferation with viability of over 60%, indicating low toxicity. Nevertheless, the viability of HDFs decreased as the concentration of TQ increased. In this study, the efficacy of TQ, the principal bioactive component of NS, as an antibacterial agent was evaluated. The antibacterial activity was assessed using the well diffusion method to measure the zones of inhibition and the broth microdilution assay to determine the MICs. Regarding Gram-positive bacteria, the most significant inhibitory effect against *Bacillus cereus* was observed with concentrations of TQ at 1 and 0.5 mg/mL. In contrast, for *Staphylococcus aureus*, a TQ concentration of 0.0156 mg/mL displayed the greatest zone of inhibition. Both concentrations exceeded the inhibitory effect of gentamicin by a substantial margin. In relation to Gram-negative bacteria, like the findings for the Gram-positive bacteria, a TQ concentration of 0.0156 mg/mL showed the greatest zone of inhibition against *Escherichia coli*, significantly larger than that of gentamicin. On the other hand, for *Pseudomonas aeruginosa*, the greatest inhibitory effect was observed at a TQ concentration of 1 mg/mL. Nonetheless, the inhibitory effect was significantly lower than that of the positive control. Based on the zone of inhibition data, TQ demonstrated a broad-spectrum efficacy against both Gram-positive and Gram-negative bacteria. The MIC of TQ, determined through the broth microdilution assay, was found to be 0.0625 mg/mL for both Gram-positive bacteria and 0.125 mg/mL for both Gram-negative bacteria. TQ exhibited inhibitory effects against both Gram-positive and Gram-negative bacteria, with a lower MIC value for Gram-positive bacteria and a higher MIC value for Gram-negative bacteria, consistent with those observed by Goel and Mishra (2018) [38]. Based on the dose–response analysis, zone of inhibition data, and MIC values, TQ concentrations of 0.05% and 0.1% were chosen for subsequent assessment. Control groups were employed for comparison, consisting of non-crosslinked and non-TQ-incorporated groups.

Grossly, all biomatrices were white in color, regardless of the crosslinking status. One of the keys to successful biomatrix fabrication from different biomaterials is the maintenance of the original properties. This is important to prevent any rejection in future clinical conditions [39]. Therefore, chemical characterization was carried out to guarantee the preservation of the original structure of the biomaterials. FTIR analysis was performed to identify the functional groups of the biomatrices. The FTIR spectra of all biomatrices showed similar absorbance peaks, corresponding to Amide A (3500–3100 cm^−1^), Amide I (1634–1618 cm^−1^), Amide II (1551–1531 cm^−1^), and Amide III (1285–1231 cm^−1^). The absorption vibration between 3500 and 3100 cm^−1^, referred to as Amide A, is associated with N-H stretching vibrations [40]. The Amide I band, which ranges from 1634 to 1618 cm^−1^, is related to the carbonyl (C=O) stretching vibration in the amide groups and is the most important factor for exploring the secondary structure of proteins [41]. The absorption vibration between 1551 and 1531 cm^−1^, referred to as Amide II, corresponds with N-H bending vibrations coupled with stretching C-N vibrations [39]. The last region, Amide III, whose band ranges from 1285 to 1231 cm^−1^, is related to C-O vibrations [39]. The biomatrix showed obvious absorption peaks, which indicated that the product was fabricated from protein [41]. After GNP crosslinking, there was no major shift dominant in the FTIR spectra. EDX was performed to identify the elements of the biomatrices that could be further analyzed to determine homogeneity [42]. The examination of the electron images of the biomatrices revealed three primary elements, which were carbon (58.9–71.2%), oxygen (16.7–23.1%), and nitrogen (11.6–15.0%). These three elements are the major components of collagen, gelatin, and elastin proteins. Among all biomatrices, CollaGee had the highest carbon percentage, with a value of 71.2%. The non-crosslinked biomatrices had a higher carbon percentage compared with the respective crosslink biomatrices. These results are consistent with those of a study by Salleh et al. (2022), where non-crosslinked bioscaffolds of both the single collagen layer and the dual layer of collagen and gelatin nanocellulose had a higher carbon percentage compared to the crosslinked bioscaffolds [42]. Comparing oxygen, the crosslinked biomatrices had a higher oxygen percentage compared with their respective non-crosslinked biomatrices. Although TQ corporation resulted in slightly lower carbon percentages and slightly higher oxygen percentages, nevertheless, there were no major differences in the percentages of these elements among the biomatrices. Sulfur was found in trace amounts, which might have been due to the usage of DMSO to dissolve the TQ. Overall, FTIR and EDX analyses demonstrated no major modifications or elements found after GNP crosslinking. Even while the biomatrix did not change the fundamental native chemistry, it might, nevertheless, have reacted differently with HDFs.

A biomatrix must be biocompatible to allow cell migration, attachment, and proliferation within the 3D biomatrix [43]. Investigating cytocompatibility is crucial to understand how different biomaterials and bioactive compounds affect cells. Cell viability is defined as the ability of cells to perform specific functions and be capable of division [44]. Cell viability on the biomatrices was assessed using an MTT assay. The principle of the MTT assay is the reduction of water-soluble yellow tetrazolium salt into water-insoluble blue/magenta formazan crystals. The concentration of dissolved formazan crystals can be measured using a spectrophotometer [44]. The percentage of cell viability for all treated groups at days 1, 3, and 7 showed more than 80% viability, indicating that CollaGee biomatrices incorporated with TQ dissolved in DMSO, along with GNP dissolved in EtOH, were cytocompatible and not cytotoxic to HDFs. The cell attachment assay showed that the biomatrices provided a good attachment surface for HDFs, with cell attachment of more than 70%. Biomatrices made from proteins such as collagen, gelatin, and elastin have received a lot of attention up to this point. The surface of the material promotes direct interaction between the materials and host cells. Consequently, more hydrophilic materials tend to interact more effectively with cells. The hydrophilicity was increased by collagen peptides, which could facilitate the interaction of the cells with the scaffolding matrix [45].

LIVE/DEAD^®^ assay and SEM analysis were utilized to examine the cytocompatibility of the biomatrices towards HDFs. Fluorescence images of the LIVE/DEAD^®^ assay revealed homogeneous cell distribution and good cell viability. After 24 h, the HDFs changed from round to spindle-shaped. This spindle-shaped morphology of HDFs indicated that the cells attached and proliferated in the 3D biomatrix [46]. This good viability indicated that TQ incorporation in the CollaGee biomatrices along with GNP crosslinking was completely safe for the HDFs. According to SEM examination, the HDFs seeded onto the biomatrices revealed round to spindle-shaped morphology, with good attachment and an extended cytoskeleton. A healthy HDF, as observed via SEM, was characterized by diverse morphological shapes, featuring temporary projections of pseudopodia and the secretion of matrices (whitish granules) in the surrounding area. ICC was used to identify Col-I, vinculin, and α-SMA in the biomatrices. Fluorescence staining with DAPI and the secondary antibody clearly revealed the expression of Col-I, vinculin, and α-SMA within the 3D structure. HDFs showed morphologies ranging from round to spindle-shaped, with uneven distribution. One of the advantages of using CLSM is the ability to reconstruct and visualize the distribution of the cells within 3D biomatrices. ICC analysis observed on CLSM revealed cellular organization and connections between the fibers in the biomatrices.

The degradation kinetics of CollaGee ASSs are influenced by the initial size and thickness of the biomatrices. For a standard ASS measuring 1 cm × 2 cm × 2 cm (width × length × height), enzymatic biodegradation is expected to occur within four days. TQ is anticipated to aid in preventing infection during application and may facilitate wound healing in full-thickness skin wounds. Since full-thickness wounds require longer healing times compared to superficial wounds, it is predicted that ASSs will need to be applied at least twice. The thermal stability of the product is essential for its long-term storage. The crosslinking of the ASS demonstrated optimal thermal stability, allowing it to withstand dry storage in any season and maintain its functionality upon application. Nevertheless, further investigations are necessary to explore the long-term stability and performance of CollaGee. In follow-up studies, the antibacterial properties of the TQ-incorporated CollaGee biomatrix need to be evaluated to ensure the antibacterial activity of the product. It should be noted that the potential adverse effects, including immunoreactivity with TQ, may arise. Therefore, in-depth in vivo immunogenicity testing and preclinical and clinical studies should be conducted, investigated, and truly evaluated before considering medical applications.

## 5. Conclusions

In vitro assessments of GNP-crosslinked TQ-incorporated CollaGee biomatrices were conducted. The findings revealed that the incorporation of TQ demonstrated no toxicity towards HDFs. Comparing both TQ concentrations, we concluded that CollaGee0.05%_GNP was the better choice for future analysis, as 0.05 mg/mL of TQ was capable of inhibiting the growth of both Gram-positive and Gram-negative bacteria, with good cytocompatibility and less toxicity. Progressing to in vivo evaluation on animal models is worth pursuing to assess the rate of healing of full-thickness skin wounds. ASSs consisting of collagen, gelatin, and elastin; incorporated with TQ; and crosslinked with GNP could be used for the future rapid treatment of full-thickness skin wounds.

## Figures and Tables

**Table 1:** MICs of TQ against *Staphylococcus aureus*, *Bacillus Cereus*, *Escherichia coli*, and *Pseudomonas aeruginosa*.

Antibacterial Agents	*Staphylococcus aureus* (mg/mL)	*Bacillus cereus* (mg/mL)	*Escherichia coli* (mg/mL)	*Pseudomonas aeruginosa* (mg/mL)
Gentamicin	0.0156	0.0156	0.0156	0.0156
TQ	0.0625	0.0625	0.125	0.125

**Table 2:** EDX analysis of TQ-incorporated CollaGee biomatrices.

Groups	Carbon (%)	Oxygen (%)	Nitrogen (%)	Sulfur (%)
CollaGee	71.2	16.7	12.0	Nil
CollaGee_GNP	70.4	17.4	12.2	Nil
CollaGee0.05%	67.3	17,5	12.5	2.7
CollaGee0.05%_GNP	58.9	23.1	15.0	2.9
CollaGee0.1%	64.6	19.0	12.2	4.2
CollaGee0.1%_GNP	63.8	19.7	11.6	4.9

## Data Availability

The data presented in this study are available upon request from the corresponding author.
